# Assessing olfactory functions in patients with Barth syndrome

**DOI:** 10.1371/journal.pone.0187619

**Published:** 2017-11-03

**Authors:** Michele Dibattista, Simona Lobasso, Sebastiano Stramaglia, Angela Corcelli

**Affiliations:** 1 Department of Basic Medical Sciences, Neuroscience and Sense Organs, University of Bari Aldo Moro, Bari, Italy; 2 Department of Physics, University of Bari Aldo Moro, Bari, Italy; 3 INFN, Sezione di Bari, Bari, Italy; 4 IPCF-CNR Sezione di Bari, Bari, Italy; Instituto Cajal-CSIC, SPAIN

## Abstract

Barth syndrome is a rare X-linked disease affecting less than 200 individuals worldwide. Several comorbidities have been associated with the pathology and, among those, cardiac myopathy and neutropenia are the most life threatening. The appropriate nutritive support is important to sustain the everyday life of Barth syndrome patients given the chronic fatigue they experience. Since they often prefer salty and fried food, and avoid vegetables and fruits, their eating habit and food preferences do not always provide the proper amount of vitamins and amino acids. It has been indeed reported that Barth syndrome patients have altered taste sensitivity. As olfaction also contributes to food consumption and flavor perception, we decided to investigate their olfactory abilities using the “Sniffin’ sticks’ extended test”. We found no significant difference in any of the tested olfactory abilities between the group of Barth syndrome patients and the healthy controls. In summary, altered food preference of Barth boys could not be easily explained with an altered olfactory perception.

## Introduction

Barth syndrome (BTHS) is a life-threatening, X-linked recessive disease characterized by infantile onset of cardiac and skeletal myopathy, neutropenia, growth delay, and increased urinary excretion of 3-methylglutaconic acid [1–3]. The syndrome is considered an extremely rare disease with currently less than 200 known living patients worldwide (data courtesy from Barth Syndrome Foundation, USA). Heart failure or severe bacterial infections due to neutropenia by three years of age are currently the most common cause of death [3].

BTHS is caused by loss-of-function mutations of the tafazzin (*TAZ*) gene, located at Xq28 [4], which then lead to the abnormal remodeling of the mitochondrial phospholipid cardiolipin, essential for mitochondrial functions [5,6]. An increased level of the phospholipid monolysocardiolipin and a lower level of mature cardiolipin are often observed in mitochondria of BTHS patients [7–9]. As cardiolipin plays an important role in maintaining correct mitochondrial membrane structure, abnormal mitochondrial morphology and variable energy metabolism dysfunctions have been described in patients with severe BTHS cardiomyopathy [3].

The clinical signs of the disease vary according to the mutation in the *TAZ* gene carried by the subjects thus making the syndrome quite heterogeneous in its manifestation. This makes it difficult to evaluate the effect of the comorbidities, except for the most dangerous neutropenia and cardiomyopathy [3].

Nowadays, early diagnosis and modern treatments have significantly improved the survival rate of patients, which is related to a special attention on their quality of life. Improving survival rate demands more attention on the quality of life of the patients. For example, an adequate diet should be important to provide the right amount of calories and vitamins, but nutrition can be a complicated issue for parents, caregivers and BTHS patients themselves [10]. It seems that BTHS patients have altered food preferences preferring food with strong flavors. They crave salty, cheesy or spiced food, and their food preferences are often different from those of other family members. According to Reynolds et al. (2015), BTHS boys are classified as “supertasters” based on their score of the phenylthiocarbamide (PTC) test. This test is used to explore the genetic polymorphisms of the bitter taste receptor gene *(TAS2R38)* based on the bitterness rating of a chemical, the PTC. Subjects having a polymorphism in the *TAS2R38* gene, rate PTC as extremely bitter and were called supertasters. Thus, the supertaster condition of BTHS patients could be correlated with their lower intake of vegetables and citrus fruits, as well as greater fear of trying new food [11,12]. These habits define them as picky eaters and/or neophobic [12]. In addition, it has been reported by caregivers that patients often display a hypersensitive gag reflex elicited by actual foods and food odors. This anecdotal evidence prompted us to investigate the olfactory abilities of BTHS patients to better understand their olfactory perception, as olfaction is important for flavor perception, with odors playing important roles in anticipation and food consumption [13].

To explore the BTHS patient olfactory abilities we used the Sniffin’ Sticks Test.

## Material and methods

### Subjects

All subjects of this study were recruited at the Barth Syndrome 8^th^ International Scientific, Medical & Family Conference, in Florida (USA) in 2016. We recruited 30 healthy controls and 32 patients with ages spanning from 6 to 34 years old. In our testing, average age was not significantly different between the two groups of subjects (Ctrl = 16.3 ± 8 and BTHS = 16.5 ± 8, p = 0.9, see Table 1). All BTHS subjects and controls were males because of the X-linked nature of the disease. The location and the event itself gave us a great opportunity, because BTHS, being an extremely rare disease, make it difficult to recruit patients for testing. Although the conference took place in the United States, patients and entire families (with also healthy relatives) came from different countries.

**Table 1:** Sniffin’ Sticks olfactory test comparing Barth syndrome patients (BTHS) vs control (Ctrl).

	Ctrl	BTHS	p values
N	32	30	-
*Age*	16.3(8.0)	16.5(8.0)	0.90^a^
*Threshold*	9.3(4.3)	8.3(3.5)	0.22^a^
*Discrimination*	11.1(2.3)	10.7(2.9)	0.62^b^
*Identification*	11.4(2.0)	12(1.9)	0.34^a^

Values are mean (SD)

^a^Mann Whitney U-test.

^b^Student t-test.

We tested patients and control subjects in the same room and in the same experimental conditions (temperature, humidity, etc.).

All aspects of the study were approved by the Institutional Review Board (IRB n. 5069/2016 by Ethical committee of the Azienda Ospedaliera-Universitaria “Consorziale Policlinico”) and were compliant with the Declaration of Helsinki. On the basis of the approval of our study by the Italian IRB, we have been invited by the Barth Syndrome Foundation Scientific and Medical Advisory Board to perform the olfactory test during the 2016 Family Conference. Prior to testing, all parents or legal guardians provided written informed consent, and all children provided their verbal assent to participate. Participants were informed that they could discontinue participation at any time. They also filled out a medical questionnaire where they reported Barth related comorbidities and any other problems, specifically related to olfactory dysfunction and/or trauma. None of the subjects reported any nasal pathology and all subjects that started the olfactory test completed the three different subtests.

### Sniffin’ Sticks test

The Sniffin' Sticks Test (Burghart Medizintechnik Inc. Wedel, Germany) was used to investigate olfactory performance.

This test consists of using pen-like odor dispensing devices with the felt tip containing odors instead of ink. It tests three olfactory functions: odor threshold, odor discrimination and odor identification [14]. The tests were administered following manufacturer instructions. Pen caps were removed only during odor presentation and positioned approximately 2 cm for 3 s, in front of both nostrils. The pens were presented one at a time at an interval of about 30 s to avoid adaptation.

#### Threshold testing

The subjects were first asked if they were familiar with the rose odor (2-phenyl ethanol) and we let them smell the pen containing the highest odor concentration. Then, using a triple-forced-choice paradigm, three pens (triplets) were randomly presented to each subject; two contained the solvent (without odor) and one contained the rose odorant in different dilutions. The task was to detect which pen contained the odorant. The triplets’ presentation started with the lowest concentration of the odorant. If the subject did not identify the pen containing the odor, then the next triplet with the next higher concentration was presented. If the subjects correctly identified the pen containing the odor, then the next triplet with a lower concentration was presented (staircase reversal method). Once six reversals were reached, the average of the last four reversals was used to estimate the detection threshold. As sixteen odor dilutions were present in the pens, the score ranged between 1 and 16.

#### Discrimination testing

This test was administered following a forced-choice paradigm, where two pens had the same odor and a third contained a different one; subjects were asked to “discriminate” which pen smelled differently. Because 16 triplets were tested the subjects’ scores ranged from 0 to 16.

#### Identification testing

Odor identification was assessed for 16 common odors. Using again a forced-choice paradigm, subjects were presented one odor, which they had to identify from a choice of four odors represented as pictures.

#### TDI score

The three scores within each test were converted to Z-scores and then summated to obtain TDI score.

### Statistical analysis

To test for the normality of data distribution we performed Shapiro-Wilk normality test; then classical descriptive statistics with the appropriate statistical test (either Mann-Whitney U-test or t-test, Table 1) were used to test differences between the two subject samples (BTHS patients and healthy controls). Descriptive statistics and plots were made in R environment (R Core Team, 2012).

Data were then submitted to unbalanced ANOVA with the condition (Barth or Control) and Neutropenia as factors between groups, as well as age as a covariate; Matlab 2016 was used to perform the analysis.

## Results

We used Sniffin’ Sticks test to assess olfactory abilities of 30 BTHS patients and compare them to 32 healthy subjects (Ctrl). Descriptive statistics are shown in Table 1. Our data showed no significant differences in threshold, discrimination and identification scores between controls and BTHS, suggesting that patients performed as healthy subjects.

Furthermore, we transformed the score of each olfactory test to Z-scores and then summated the values to obtain TDI scores. The TDI scores for the two groups were not different (control = 0.051 (2.23) and for BTHS = -0.054 (2.42), p = 0.93 calculated by Mann Whitney U-test).

**Fig 1:**
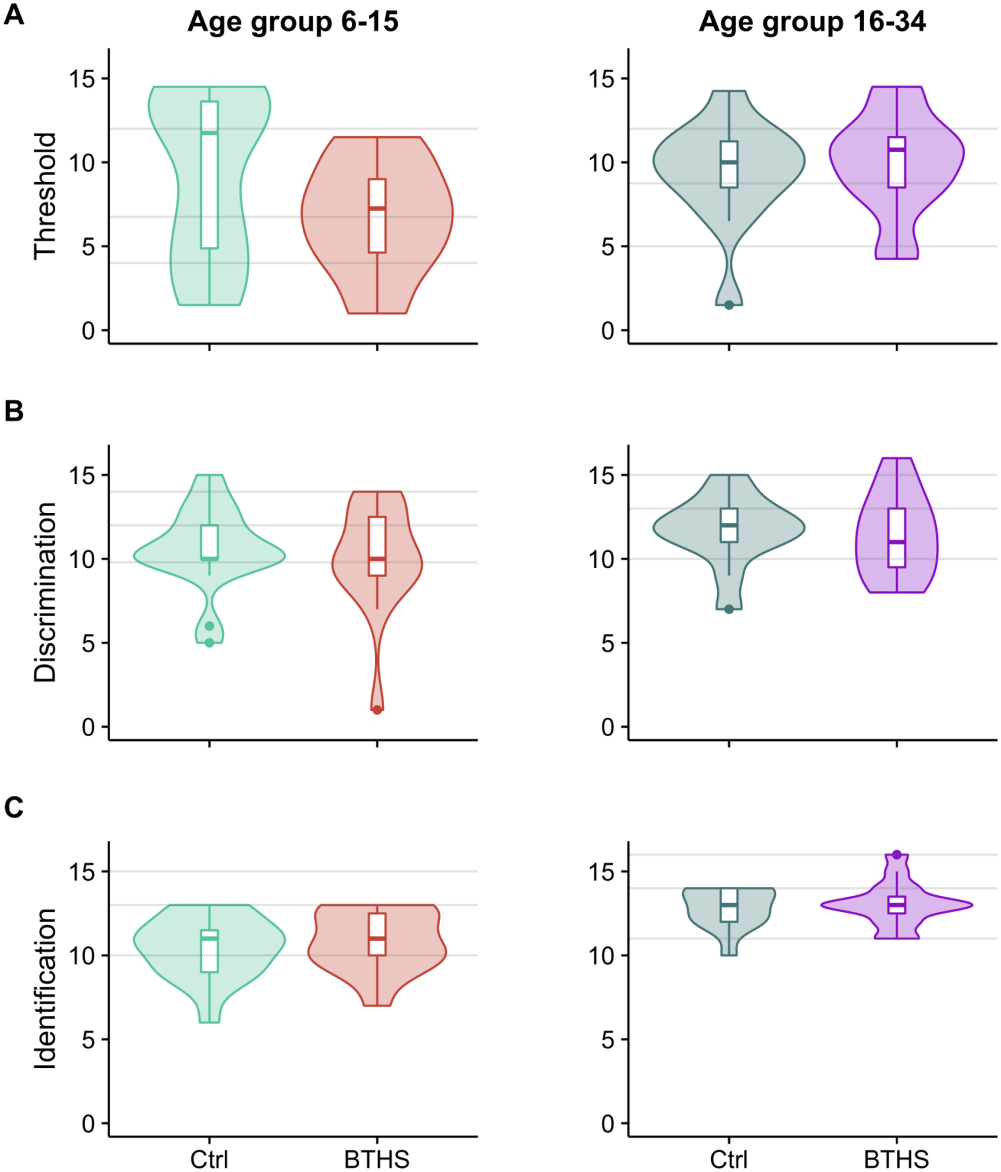
Violin plots for threshold (A), discrimination (B), and identification (C) scores. The violin plots showing the Sniffin’ Sticks scores: left side showing scores for control and BTHS patients aged between 6 to 15 years and right side showing scores for subjects aged between 16 to 34 years. The width of each violin is proportional to the number of participants scoring that value. Boxplots indicating median, 95^th^, 75^th^, and 10^th^ percentiles are shown inside the violin. Grey lines represent the normative data from Hummel et al. 2007 [15] for the same age groups in control population (median, 95^th^ and 10^th^ percentiles).

Both groups of our study were of ages ranging from 6 to 34 years. In Table 2, we show the descriptive statistics of Sniffin’ sticks tests obtained by dividing our subjects into four groups: two age groups for controls and two for BTHS, children from 6 to 15 and young adults from 16 to 34 years of age, following the same grouping criteria as in Hummel et al. 2007 [15]. This would allow us to compare our data with the normative scores from data collected from over 3000 healthy subjects published by Hummel and collaborators in 2007. Violin plots for threshold, discrimination and identification tests are shown in Fig 1. Grey lines in the violin plots represent the normative data from Hummel et al. 2007 [15] for the same age groups in control population (median, 95^th^ and 10^th^ percentiles).

**Table 2:** Sniffin’ Sticks test comparing Barth syndrome patients (BTHS) vs controls (Ctrl), divided into age groups.

	Age 6–15		Age 16–34	
	Ctrl	BTHS	Ctrl	BTHS
**N**				
	19	15	13	15
**Threshold**				
mean(SD)	9.2(4.9)	6.7(3.3)	9.4(3.1)	9.9(3.0)
median	11.7	7.2	10.0	10.7
[lower ci-upper ci]	[6.9–11.5]	[4.9–8.5]	[7.6–11.2]	[8.3–11.5]
**Discrimination**				
mean(SD)	10.6(2.4)	10.1(3.3)	11.7(2.0)	11.4(2.5)
median	10.0	10.0	12.0	11.0
[lower ci-upper ci]	[9.5–11.7]	[8.3–11.8]	10.5–13.0]	[10.0–13.0]
**Identification**				
mean(SD)	10.5(1.8)	10.9(1.8)	12.8(1.2)	13.1(1.4)
median	11.0	11.0	13.0	13.0
[lower ci-upper ci]	[9.6–11.3]	[9.9–11.8]	[12.1–13.5]	[12.3–13.8]

Results are reported as mean, standard deviation (SD), median and 95% confidence intervals ([lower ci-upper ci]).

Since age related differences in olfactory tests are well documented by using a variety of different methods [15,16], we decided to consider age as a covariate of no interest in our statistical analysis.

Although, once corrected for age, no significant differences were detected in the *Threshold* tests (*F*_*(1*,*59)*_ = 0.4, p = 0.55), it is worth notice that in the age group 6–15 BTHS score distribution was constantly below that control and BTHS children showed a tendency toward lower threshold values (Fig 1A, left and Table 2). Also, no significant differences were found in *Discrimination* (*F*_*(1*,*59)*_ = 0.15, p = 0.7, see Fig 1B) and *Identification* (*F*_*(1*,*59)*_ = 3.1, p = 0.08, Fig 1C) and TDI score (*F*_*(1*,*59)*_ = 0.05, p = 0.82) with our statistical model.

In summary, although different in their distributions (at least for the threshold score), we could not detect any significant difference between BTHS and control in the olfactory tests.

**Fig 2:**
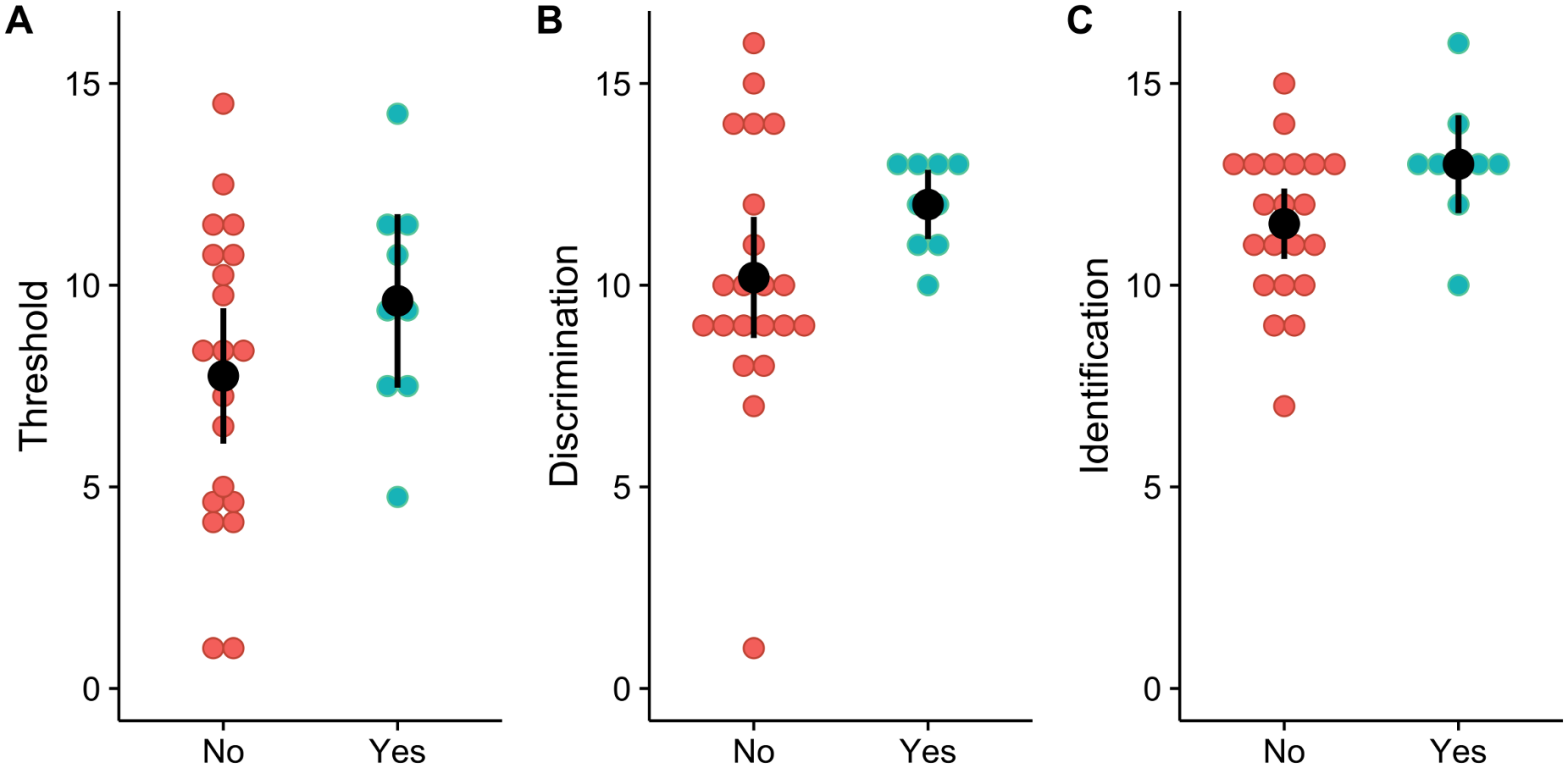
Test scores of BTHS patients under treatment for neutropenia and patients without medication. Dotplots of Threshold (A), Discrimination(B), and Identification (C) for BTHS subjects without and with treatment for neutropenia (No, coral circles and Yes, cyan circles). Black circles and bars represent mean and 95% confidence interval for each score in each group.

In addition, we evaluated the effect of comorbidities on the performance of Sniffin’ Sticks test. Most BTHS patients present persistent, intermittent or cyclical neutropenia, although normal neutrophil counts can be observed over long periods in some subjects [3]. The contemporaneous presence of severe neutropenia, cardiomyopathy, and general mitochondrial dysfunction may increase the probability of death due to severe bacterial sepsis. In our study 10 BTHS patients were Neutropenic under medication with Granulocyte Colony Stimulating Factor (G-CSF) and we decided to investigate whether those factors could affect the olfactory test scores (see Fig 2). Although none of the scores were significantly affected by introducing neutropenia in our statistical model, we observed that 6 out of 20 (34%) BTHS patients without neutropenia and only 1 out of 10 (10%) of BTHS with neutropenia scored below or equal to 10 (Fig 2, panel C) which is considered as the value that separates hyposmic from normosmic subjects (10^th^ percentile value from ref. [15]) thus suggesting a possible effect of Barth comorbidity on the identification ability.

## Discussion

Flavor is a complex mixture of sensations among which olfaction is a major player. It has different important functions that are usually underestimated in modern life [17]. Food preferences and/or eating behavior are driven by olfactory experience. Precisely, in the early life, this experience is critical for the formation of individual food preferences and for healthy habits [18–20], especially when feeding problems can lead to vitamin deficiencies [21]. A further role of olfaction is the avoidance of spoiled food: one can smell dangerous food before it comes in direct contact with the oral cavity, the gut and other related organs [22].

Besides smelling volatile molecules in the environment around us, olfaction plays a role in flavor perception through its retronasal pathway. Once the food is in the oral cavity, mastication liberates volatile molecules that through the nasal pharynx reach the olfactory epithelium, located in the nasal cavity, leading to the predominant flavor sensation [23,24].

It has been previously reported that BTHS patients might have taste sensitivity issues but it was not clear whether they have olfactory related problems [12]. To our knowledge, our work is the first to address directly the olfactory abilities of BTHS patients with a standardized method: the Sniffin’ Sticks Test. Here we observed that subjects affected by BTHS performed the test similarly to healthy controls.

When we sought to investigate the effect of Neutropenia, we found in neutropenic patients (33% of BTHS subjects) a tendency to improve the scores that in our opinion is worth to discuss. By comparing our data with the normative values published by Hummel T. et al 2007 [15] and considering the score below which a subject can be hyposmic (10^th^ percentile rule) none of BTHS patients with neutropenia and under G-CSF felt under that value in the identification test. Neutropenia, as BTHS associated comorbidity is cyclic and it consists of a low count of neutrophils thus making the young BTHS patients susceptible to infections. To our knowledge, there are no data in the literature about whether neutropenia can influence olfactory perception.

Taking G-CSF helps to increase the absolute neutrophil counts [3]. Interestingly, several studies revealed the hematopoietic G-CSF as a neurotrophic factor playing a role in neuroprotection and neuroregeneration [25,26]. It has been shown that G-CSF passes the intact blood-brain barrier and that both G-CSF and its receptor are widely expressed by neurons in the CNS, including neurons in the olfactory bulb namely the mitral cells [25].

The G-CSF receptor is also expressed by adult neural stem cells [25]. It is well known that the olfactory neuroepithelium can generate new neurons from a population of precursor stem cells present in the basal cell layer. The newly formed primary olfactory neurons give rise to axons that reach the olfactory bulb [27]. In this scenario, the presence of G-CSF could play a role in apoptosis and proliferation of olfactory neurons, as demonstrated in other neuronal cell types [25].

In addition, it seems that the administration of G-CSF was able to increase the engraftment of transplanted bone marrow-derived cells into the olfactory epithelium in mice, probably creating the right microenvironment for regeneration [25,28]. It would be interesting in the future to explore the effect of the G-CSF on human olfactory epithelium and sensory abilities.

Blood related syndromes, like the von Willebrand (vW) type 3 disease, seem to affect the olfactory ability of patients, even though it is not yet clear whether those are anosmic [29] or not [30], and whether treatments for vW syndrome improve patients’ olfactory performance.

In vW syndrome, though, the gene encoding for the calcium-activated chloride channel, the *TMEM16B* [29,31], expressed in the olfactory epithelium and important for the signal transduction, may be mutated. Furthermore, the vW factor type A domain of the calcium-activated chloride channel regulators (CLCA) is necessary for the activity of some members of the family of TMEM16 channels [32,33]. This suggests that the vW factor itself may modulate elements of the olfactory signaling cascade (*i*.*e*. the TMEM16B channel and/or CLCAs).

In conclusion, by conducting the Sniffin’ Sticks extended test on BTHS patients we did not observe any significant differences in their olfaction. Further experiments are needed to better understand sensory abilities of patients affected by BTHS. We suggest that altered food preference of BTHS patients may derive from complex multisensory interactions and not just from altered olfactory perception.

## Supporting information

S1 File Sniffin’ Sticks test raw data. (XLSX)
